# SOX6-MAP4K4 pathway induces autophagy and contributes to the reduced chemosensitivity of cervical cancer

**DOI:** 10.1080/27694127.2022.2040917

**Published:** 2022-03-17

**Authors:** Hongxin Huang, Jie Wang

**Affiliations:** Department of Microbiology & Infectious Disease Center, School of Basic Medical Sciences, Peking University Health Science Center, Beijing, 100191, China

**Keywords:** Autophagy, cervical cancer, chemotherapy, cisplatin, MAP4K4, SOX6

## Abstract

Some cervical cancer patients show poor response to neoadjuvant chemotherapy. Although it has been demonstrated that macroautophagy/autophagy contributes to the poor chemosensitivity, the underlying mechanism has not yet been fully clarified. In this autophagy punctum, we discuss our recent findings about the mechanism of SOX6-induced autophagy and its potential significance in the platinum-based chemotherapy of cervical cancer.

Neoadjuvant chemotherapy followed by radical hysterectomy and lymphadenectomy is an effective treatment option for locally advanced cervical cancer; however, nearly 40% of cervical cancer patients show poor response to neoadjuvant chemotherapy. At present, the underlying mechanism of poor response and how to increase the response to platinum-based chemotherapy in cervical cancer have not yet been fully clarified and solved, respectively. In Huang et al. [1], our findings for the first time uncover the underlying mechanism and clinical significance of SOX6 (SRY-box transcription factor 6)-mediated autophagy in mediating the chemosensitivity of cisplatin to cervical cancer, and shed new light on the usage of MAP4K4 (mitogen-activated protein kinase kinase kinase kinase 4) inhibitor or autophagy-specific inhibitor for sensitizing cervical cancer cells to platinum-based chemotherapy.

First, we found that SOX6 promotes autophagosome formation and autophagic flux in cervical cancer cells, depending on its HMG domain. To investigate the underlying mechanism responsible for SOX6-induced autophagy, microarray and RNA-sequencing analyses were performed, and *MAP4K4* gene was identified as the potential target gene of SOX6. Further, we confirmed that SOX6 can upregulate both mRNA and protein levels of MAP4K4, which is dependent on its HMG domain. Further, dual-luciferase assay and chromatin immunoprecipitation combined with PCR (ChIP-PCR) assay confirm that SOX6 can enhance the transcriptional activity of the *MAP4K4* gene promoter through binding of its HMG domain with the double-binding sites located at 59-77 and 430-444 bp upstream of the transcriptional start site (TSS) of the *MAP4K4* gene. Meanwhile, the SOX6-induced autophagy is remarkably reduced when the expression of endogenous MAP4K4 is knocked down by the *MAP4K4*-specific small interfering RNAs (si*MAP4K4*) or inhibited by the MAP4K4-specific inhibitor PF-06260933. The above results suggest that the *MAP4K4* gene is the direct target gene of SOX6 and mediates the SOX6-induced autophagy. Furthermore, we found that MAP4K4 mediates the SOX6-induced autophagy through activating the MAPK/ERK pathway and inhibiting the PI3K-AKT-MTOR pathway.

To further investigate whether the SOX6-induced autophagy affects the efficacy of cisplatin, the effect of SOX6 in the cisplatin-induced apoptosis of cervical cancer cells was analyzed. The result revealed that the SOX6-induced autophagy can reduce the chemosensitivity of cervical cancer cells to cisplatin *in vitro* and *in vivo*. In turn, we found that both the level of endogenous SOX6 protein and its induced autophagy are significantly increased under cisplatin treatment, and accompanied by an increased level of MAP4K4, activation of the MAPK/ERK pathway, and inhibition of the PI3K-AKT-MTOR pathway in cervical cancer cells. Meanwhile, compared to HeLa cells, the lower level of endogenous SOX6 protein contributes to the higher sensitivity of CaSki cells to cisplatin treatment, indicating the possibility that the endogenous SOX6 upregulated by cisplatin may in turn reduce the sensitivity of cervical cancer cells to cisplatin treatment. This possibility was further confirmed by the increasing response to cisplatin when the *SOX6* gene was knocked out in HeLa cells. Moreover, the role of SOX6-induced autophagy was explored in cervical cancer patients who underwent neoadjuvant chemotherapy, that is, the routine platinum-based chemotherapy. For the patients who were sensitive to chemotherapy, the protein levels of SOX6 and MAP4K4, as well as the level of autophagy are lower than those in patients who are not sensitive to cisplatin, which further demonstrates that the high level of SOX6-induced autophagy mediated by MAP4K4 may reduce the sensitivity of cervical cancer cells to platinum-based chemotherapy.

Next, we further investigated how to increase the chemosensitivity of cervical cancer cells to cisplatin, based on the existing compounds targeting the pathways of SOX6-induced autophagy. First, Baf A1, an autophagy-specific inhibitor, was used to inhibit the SOX6-induced autophagy. We found that the SOX6-reduced sensitivity of HeLa cells to cisplatin is reversed under Baf A1 treatment. Further, the chemosensitivity of HeLa cells to cisplatin can also be increased by treating with the MAP4K4-specific inhibitor PF-06260933. Accordingly, these results suggest that the chemosensitivity of cervical cancer cells to cisplatin can be increased by inhibiting SOX6-induced autophagy. Because MAP4K4 is upstream in the pathway mediating SOX6-induced autophagy, so its inhibitor may be more specific in increasing the sensitivity of cervical cancer cells to cisplatin chemotherapy.

Taken together, cisplatin can promote the expression of endogenous SOX6 and subsequently SOX6-mediated autophagy in cervical cancer cells, which may in turn reduce the chemosensitivity of cervical cancer cells to cisplatin (Figure 1). This study uncovers the mechanism of SOX6-induced autophagy and its clinical significance in the sensitivity of cervical cancer cells to cisplatin chemotherapy, which may provide possible explanations for the poor response of cervical cancer patients to platinum-based chemotherapy. More importantly, this study sheds new light on the usage of MAP4K4 inhibitors to increase the sensitivity of cervical cancer cells to platinum-based chemotherapy.

**Figure 1.**
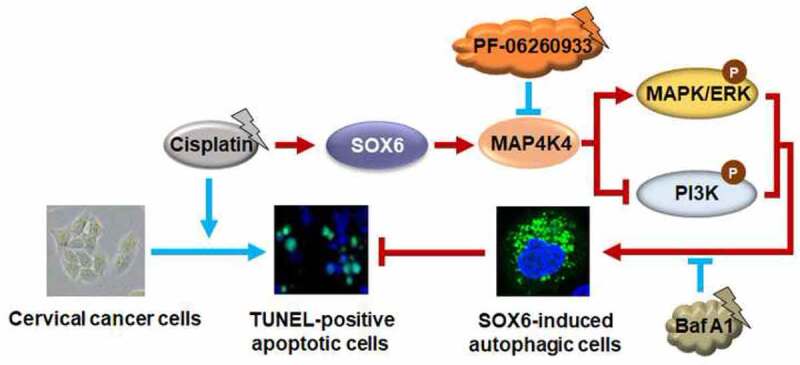
Graphical abstract on the potential mechanism and clinical significance of SOX6-induced autophagy in cervical cancer.
